# Thermodynamic, kinetic, and isotherm studies of Direct Blue 86 dye absorption by cellulose hydrogel

**DOI:** 10.1038/s41598-023-33078-2

**Published:** 2023-04-11

**Authors:** Amany G. M. Shoaib, Safaa Ragab, Amany El Sikaily, Murat Yılmaz, Ahmed El Nemr

**Affiliations:** 1 Environment Division, National Institute of Oceanography and Fisheries (NIOF), Kayet Bey, Elanfoushy, Alexandria Egypt; 2 Department of Chemical Engineering, Faculty of Engineering, Osmaniye Korkut Ata University, 80000 Osmaniye, Türkiye

**Keywords:** Environmental sciences, Chemistry

## Abstract

In this study, cellulose hydrogels were simply fabricated by the chemical dissolution method using LiCl/dimethylacetamide as a new method, and the hydrogel produced was investigated for removing Direct Blue 86 (DB86) dye from the aquatic environment. The produced cellulose hydrogel (CAH) was characterized by FTIR, XRD, SEM, and TGA analyses. The removal efficiency of DB86 dye using CAH was achieved via a batch equilibrium process. The impact of pH, time of contact, CAH dosage, starting concentration of DB86 dye, and absorption temperature were scanned. The optimum pH for absorption of DB86 dye was determined to be 2. The absorption results obtained were scanned by Langmuir (LIM), Temkin (TIM), Freundlich (FIM), and Dubinin-Radushkevich (DRIM) isotherm models (IMs) and chi-square error (X^2^) function used to identify the best-fit IMs. The CAH had 53.76 mg/g as a maximum absorption capacity (*Q*_m_) calculated from the LIM plot. The TIM was the best fitted to the CAH absorption results. Kinetic absorption results were investigated by pseudo-first-order (PFOM), Elovich (EM), pseudo-second-order (PSOM), film diffusion (FDM), and intraparticle diffusion (IPDM) models. A PSOM with a high *R*^2^ (> 0.99) accounted for the majority of the control over the absorption rate. The findings indicate that CAH can potentially remove the DB86 dye from wastewater.

## Introduction

Given the number of workers, export prices, and local production costs, the textile sector is one of the largest in Egypt. According to reports, this sector of the economy is a significant cause of stream pollution, particularly when it comes to wet procedures that include dangerous chemicals. The existence of organic pollutants like textile dyes^1,2^, pesticides^3,4^, heavy metals^5,6^, and hydrocarbons^7–9^ in the hydrosphere is of particular concern for the marine environments, coastal, and freshwater due to potentially carcinogenic and their biodegradability^10,11^.

Due to their hue, dyes, in particular, can be easily found in wastewater^11^. The most common dyes are synthetic ones utilized in different sectors, including paint, textiles, and leather^12^. Due to most pigments being carcinogenic, poisonous, and non-biodegradable, this pollution influences the health of humans and the balance of ecology^13^. It is estimated that between 10 and 20 percent of dyes discharged into water bodies are untreated, amounting to an annual average of (0.7–2.0) 105 tonnes^14^. The azo dyes take the top spot among synthetic dyes because they come in the broadest range of hues, are the largest, and have the most applications. These substances become carcinogenic when used excessively^15^. The textile industry's most widely used dyeing and printing technique is still direct dyeing. The dyeing, printing, and textile processing factories are the principal sources of homemade textile products (small factory group). Numerous industries, including textile dyeing (using 60%), plastics (10%), and paper (10%), use significant amounts of synthetic colors^16^.

There are numerous methods for handling the effluent of dyeing, but the primary ones can be summed up as absorption treatment^17–19^, advanced oxidations^20,21^, coagulation/flocculation^22^, chemical oxidation^23^ biological treatment^24^, photo-degradation^25–27^ and electrochemical management^28^.

One of these most frequently used ways is the absorption method for dye removal using activated carbon, which has a high efficiency^29,30^. However, research is now looking for less expensive and more effective adsorbent materials because of the high production and processing costs of commercial activated carbon (AC)^19,31–33^. For the treatment of water, hydrogels have become a possible substitute for activated carbon^34,35^. Polymers that have been physically or chemically cross-linked are used to create hydrogels, which expand when water is introduced. They can absorb particular contaminants through surface functionalization^34^.

The development of hydrogels as a less expensive and more environmentally friendly alternative is becoming more popular every day. Polymer materials like cellulose, alginate, chitosan, and their derivatives can be used to make hydrogels, as can synthetic materials like poly(vinyl alcohol), poly(acrylic acid), polyacrylamide, etc. Polymers from natural sources are chosen over synthetic ones in this situation because they are less expensive, biodegradable, readily available, non-toxic, intrinsically biocompatible, and meet the requirements for sustainable and renewable technical materials^36,37^. Cellulose is the most popular natural polymer precursor used for making hydrogels^38^. This is primarily because of its broad availability as an agricultural industry waste product (such as bagasse, maize cobs, rice husks, etc.) and its regenerative nature. Additionally, a range of different hydrogels with good mechanical properties can be created using cellulose thanks to the simple chemical modifications that can be made to its structure^39^.

However, due to the strong connections between the cellulose chains, cellulose barely dissolves in common solvents like methanol, ethanol, and acetone^40^. Different solvent solutions have been created; as a result of dissolving cellulose. Ionic liquids (ILs), LiCl/DMAc, NaOH/urea (or thiourea), and *N*-methylmorpholine-*N*-oxide (NMMO) are the most used solvents for cellulose^41^. Following the breakdown, cellulose chains are physically cross-linked to create a hydrogel by hydrogen bonds, ionic interactions, and physical entanglements as opposed to covalent bonds^42^. The LiCl/DMAc solvent system relies on the cellulose hydroxyl groups at the C-6 position to disrupt intermolecular hydrogen bonds. While chlorine can make hydrogen bonds with OH groups, Li^+^ cations interact ionically with free DMAc molecules. In the LiCl/DMAc solvent solution, cellulose polymers are thereby uniformly disseminated^43^.

This study aimed to produce Cellulose Hydrogel (CAH) from commercial microcrystalline cellulose by chemical dissolution using using LiCl/Dimethylacetamide, and it was examined for its effectiveness in DB86 dye elimination from water. As the DB86 dye was being eliminated from the water, variables such as the starting adsorbate concentration, solution pH, time of contact between DB86 dye and CAH, and the influence of CAH dose were studied. To ascertain the framework and maximum absorption capacity of the CAH, absorption kinetics and isotherms for Direct Blue 86 dye removal on CAH adsorbents were studied.

## Materials and methods

### Equipment and materials

**Figure 1:**
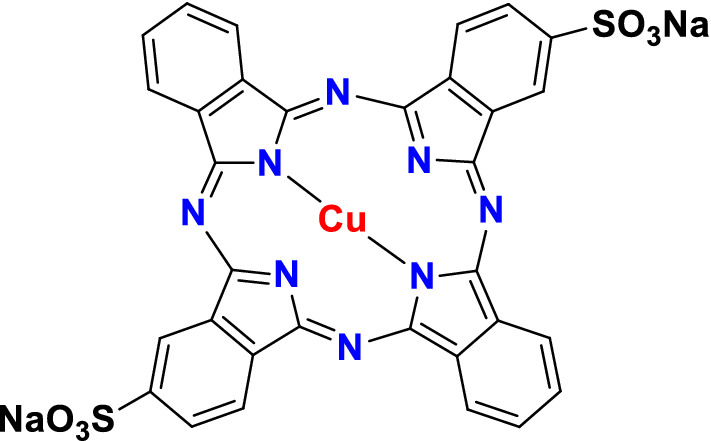
DB86 dye structure (MF: C_32_H_14_CuN_8_Na_2_O_6_S_2_) (MW: 780.17 g/mol) (C.I.74180) (Acid blue 87, Direct fast turquoise blue GL, Dragon Blue DBL 86) (CAS number: 1330–38-7).

Cellulose microcrystalline [density (0.6 g/cm^3^), sulfated ash (Max. 0.2%), purity (99.5%)] was obtained from LOBA Chemie, laboratory & Reagents fine chemical. LiCl (anhydrous) (purity 99.0% trace metals basis) was obtained Research–Lab Fine Chem Industries. *N*,*N*-Dimethylacetamide (purity 99.0%) was purchased from Advent Chembio PVT.LTD. Hydrochloric acid (HCl) (Assay 37%), NaOH, Direct Blue 86 dye (97%; CAS no.: 1330-38-7; Direct Fast Turquoise Blue GL; C.I. 74,180, Chemical formula C_32_H_14_O_6_N_8_S_2_CuNa_2_) was purchased from SD Fine-Chem Limited, Mumbai, India (Fig. 1). The reagents employed in this experiment were all of the analytical grades. Distilled water (DW) was applied to create all of the practical solutions, all the experimental work was repeated three times, and only the mean result was used throughout the analysis.

Fabrication of cellulose hydrogels.

Cellulose hydrogel (CAH) was synthesized by dissolving 1.5 g of microcrystalline cellulose (commercial) and 10 g of lithium chloride in 89 mL of *N*,*N*-dimethylacetamide. The reaction mixture was agitated at 80 °C for 24 h until gel formation. Finally, the formed was washed with DW to remove residual salt and solvent. The hydrogel obtained was freeze-dried to produce CAH.

### Characterization

Using Scanning Electron Microscopy (SEM) (JEOL-JSM-5300 LV, Tokyo, Japan) for freeze-drying under vacuum hydrogels and coating them with gold before taking pictures, the structures and morphology of the hydrogel surfaces were examined. For additional characterization, samples were dried at 50 °C for 72 h. The hydrogels' Fourier-transform infrared spectroscopy (FTIR) was captured using a platinum Attenuated Total Reflectance (ATR)-FTIR spectrometer with a wave number of V-100 VERTEX70 (400–4000 cm^–1^). The thermogravimetric analysis (TGA) was achieved under a nitrogen environment with a temp. ramp of 10 °C min^−1^ from 50 to 900 °C using a thermogravimetric analyzer (TERIOS Universal V4.5A TA Instruments). To undertake the X-ray diffraction (XRD) investigation, a Bruker X-ray diffractometer (D2 BRUKER) with Cu Kα radiation (λ = 1.540598 Å) was used. A UV–visible spectrophotometer PG instrument (model T80, United Kingdom) paired with two glass cells of 1 cm applied optical path length to measure dye concentration.

### Absorption experiments

By dissolving DB86 dye (1.0 g) in 1000 mL of DW, a DB86 dye stock solution (1000 mg/L) was created. By diluting each working solution with DW, working solutions of varied concentrations were formed. By utilizing a standard curve and a spectrophotometer at (*λ*_max_ = 615 nm), the concentration of the DB86 dye was measured. DB86 dye was eliminated using the batch equilibrium approach. The DB86 dye removed percentage by CAH was calculated. The impact of pH, time of contact, CAH dosage, beginning DB86 dye concentration, and absorption temp were investigated. The absorption isotherm, absorption kinetics, and thermodynamics parameters were examined.

Experiments of absorption occurred through the batch process. A weight of 0.15 g of CAH was added to 250 mL beakers containing 100 mL of DB86 dye solution with various starting concentrations (25–200 mg/L). The processes were performed in a shaker operated at 200 rpm at 25 ± 1 °C for 3 h. A spectrophotometer was used to quantify the DB86 dye concentrations, and Eq. (1) was used to compute the amounts of absorption.

1 $${q}_{e}=\frac{\left({C}_{0}-{C}_{e}\right) V}{M}$$

where *q*_e_ is the capacity of absorption of DB86 dye at equilibrium loaded adsorbent (mg/g), *C*_0_ and *C*_e_ represent the starting and equilibrium concentrations of DB86 dye (mg/L), *M* is the amount of CAH (g), and *V* is the solution volume (L) of DB86 dye.

By adding 0.15 g of CAH to 100 mL of 50 mg/L DB86 dye solution at a pH range from 2 to 10 adjusted using JENCO pH meter, and shaking at 200 rpm at 25 ± 1 °C, it was possible to determine the solution pH influence on elimination. A suitable amount of the reaction solution was removed after 3 h, and the remaining concentration was measured.

By adding varying amounts of 0.15, 0.30, 0.45, 0.60, 0.75, and 0.90 g of CAH into a series of flasks holding 100 mL of known DB86 dye solutions concentration and agitated at 200 rpm at 25 ± 1 °C to the equilibrium state (3 h), the CAH mass influence on DB86 dye absorption was tested.

The effects of time were investigated in a series of flasks containing 0.15 g of CAH and 100 mL of DB86 dye at different concentrations (25–200 mg/L) for 5–180 min at pH 2, agitating speed 200 rpm, and temp. 25 ± 1 °C. A suitable amount of the solution was removed at regular intervals, and the concentration was calculated.

At a temp. of 25 ± 1 °C and a pH of 2, the effects of the initial concentrations were examined by combining 0.15, 0.30, 0.45, 0.60, 0.75, and 0.90 g of CAH with 100 mL of DB86 dye solution at different beginning concentrations (25, 50, 75, 100, 150, and 200 mg/L) and shaking in a shaker running at 200 rpm. Once equilibrium had been reached, the adsorbent was removed, and the concentration remained of DB86 dye was calculated.

Chi-square error (X^2^) error functions have been explored to identify the IM that best fits the experimental equilibrium results. The X^2^ error model is shown as Eq. (2)^44^.

2 $${X}^{2}=\sum_{i=1}^{N}\frac{{({q}_{e,isotherm}-{q}_{e,calc})}^{2}}{{q}_{e,isotherm}}$$

where *N* presents the experimental data point's number.

By adding 0.15 g of CAH to a conical containing 100 mL solutions of beginning dye concentration of 50 mg/L at temperatures of 35, 40, 45, and 50 °C, the impact of temp. on the absorption process of DB86 dye was examined.

There were 3 consecutive DB86-CAH regeneration cycles carried out. In each phase, 100 mL of DB86 dye solution (100 ppm) was added to the sorbent, 0.3 g of CAH, and shaken for 3 h. After effective sorption, the DB86 dye was eluted with NaOH solution (0.1 mol/L, 100 mL) for 2 h, washed with DW, and then neutralized for reuse. Before and following the recovery phase, the removal of DB86 dye by absorption on the adsorbent CAH was compared at similar conditions: beginning solution concentration (100 mg/L) at approximately 25 °C, pH 2, and 0.3 g CAH dosage.

## Results and discussion

### Characterization

#### FTIR spectroscopy analysis

**Figure 2:**
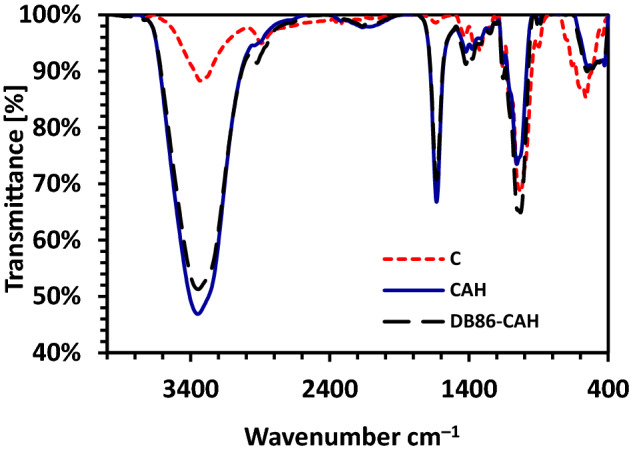
FT-IR analysis of Cellulose (C), Cellulose Hydrogel (CAH), and DB86 dye-Cellulose Hydrogel (DB86-CAH).

FTIR was applied to investigate the functional groups in cellulose powder, cellulose hydrogel, and DB86 dye-cellulose hydrogel. The FTIR spectra of C, CAH, and DB86-CAH, respectively, were reported in Fig. 2. All of these samples have absorption peaks at around 3332, 3349, and 3347 cm^−1^ which correspond to –OH stretching vibrations where the intensity of the signals increased after water absorption and decreased after DB86 dye absorption^19^. The band at 2894 cm^−1^ is assigned to C-H stretching vibration^19,45,46^. The band at 1103, 1155, 1155 cm^–1^ was assigned to the stretching vibration of C–O^47,48^ and the 1640, 1633, 1633 cm^–1^ was corresponding to the O–H bending, which was indicative to the absorption of H_2_O by high interaction between C and H_2_O^46,49,50^. The bands at 1028, 1056, and 1029 cm^−1^ correspond to –C–O– group^19,37^. The band at 898 cm^−1^ is a characteristic signal of the *β*-1,4-glycosidic linkage between glucose units in C. In addition, the peaks at 1376 cm^–1^ were associated with the bending vibration of C─H^51^.

The CAH's absorption bands were more intense and pointed than cellulose, indicating that the transition from cellulose I to cellulose II occurred following dissolution and used in the absorption of the DB86 dye^40^. The intensity of the band at 3332, 2894, 1640, and 1431 cm^–1^, which is shown in the C spectrum was increased in the CAH spectrum. This indicated that the CAH was more hydrophilic than cellulose^46^. The spectrum of CAH following DB-86 dye absorption revealed the identical peak in the spectrum that characterizes CAH with a little shift in peaks and its strength as data of absorption of DB86 dye, showing that the absorption process had taken place.

#### TGA and DTA

**Figure 3:**
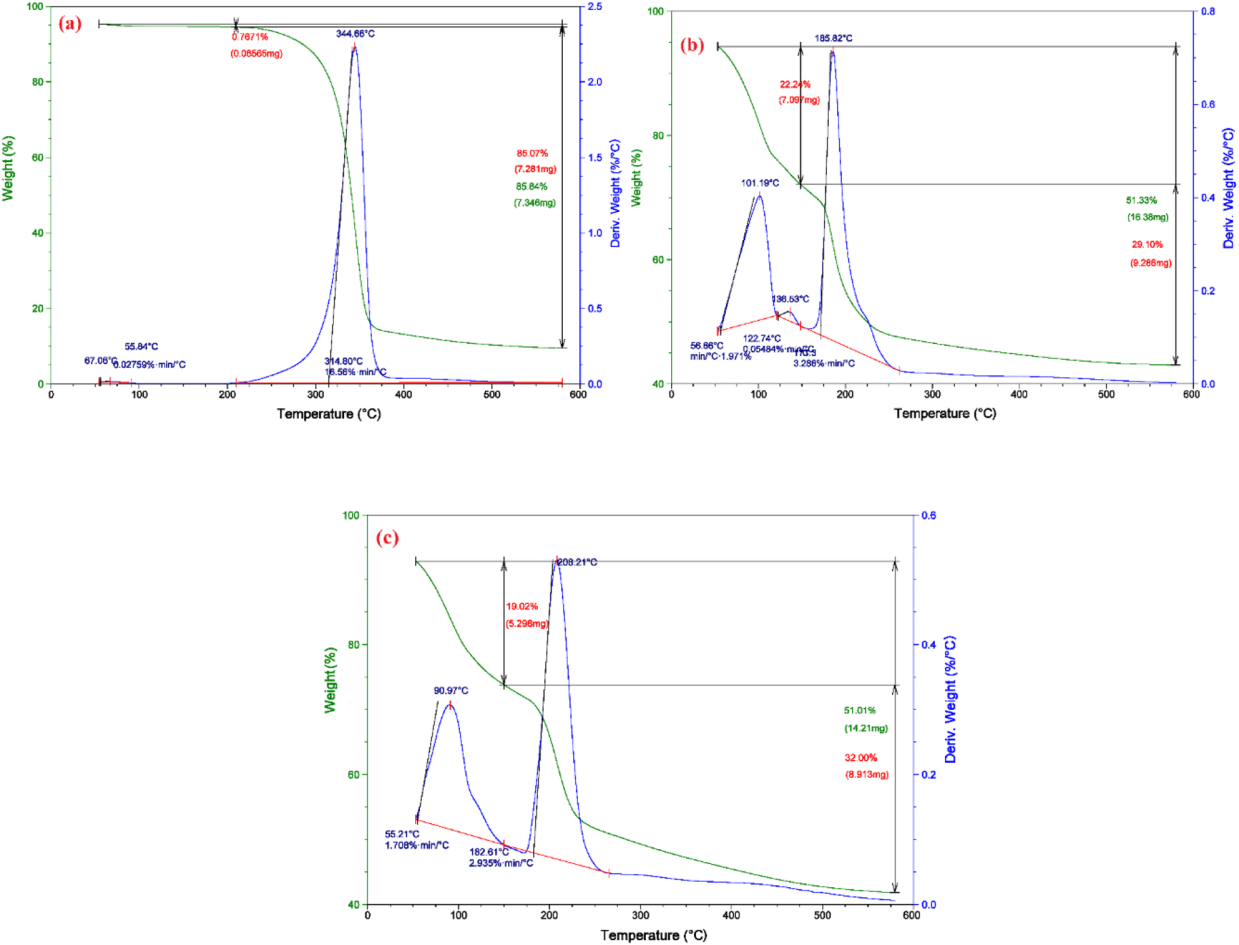
TGA, DTA of (**a**) Cellulose (C), (**b**) Cellulose Hydrogel (CAH), and (**c**) DB86 dye-Cellulose Hydrogel (DB86-CAH).

Thermogravimetric analysis (TGA) and Differential thermal analysis (DTA) of C, CAH, and DB86-CAH degradation behavior were investigated, as shown in Fig. 3, and Table 1. From thermogravimetric curves, it can be seen that powder C presents a relatively lower thermostability as it has high total weight loss. Also, thermogravimetric curves showed similar thermal behavior, with two main mass loss stages. The 1st weight loss corresponds to the evaporation of the adsorbed water from the samples with one exothermic peak from the DTA curve, and the second is assigned to the decomposition, oxidation, and combustion of cellulose with one exothermic peak from the DTA curve except for CAH it shows two exothermic peaks^52^.

**Table 1:** TGA and DTA analyses of Cellulose (C), Cellulose Hydrogel (CAH), and DB86 dye-Cellulose Hydrogel (DB86-CAH).

	C		CAH		DB86-CAH	
Total weight loss (%)	85.84		51.34		51.00	

	Weight loss stage					
	First	Second	First	Second	First	Second
*TGA*						
Temp. range (°C)	54.2–209.8	209.8–579.5	53.2–148.5	148.5–579.6	53.2–149.9	149.9–579.6
Mass loss (%)	0.7671	85.07	22.24	29.10	19.02	32.00
*DTA*						
Decomp. temp. (°C)	55.84	314.80	101.19–136.53	185.82	90.97	208.21

#### XRD studies

**Figure 4:**
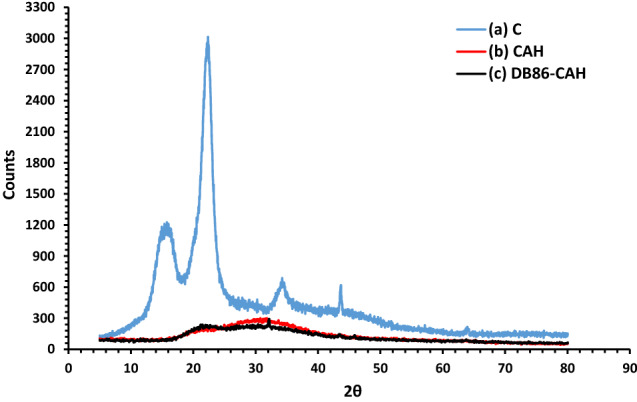
XRD analysis of (a) C, (b) CAH, (c) DB86-CAH samples.

The XRD study of C, CAH, and DB86-CAH are found in Fig. 4. The X-ray pattern of C, Fig. 4a showed four sharp, well-defined crystalline peaks observed around. 2θ of 15.005°, 22.308°, 34.395°, 43.638° indicated that the crystal form of cellulose)^53,54^. The X-ray study of CAH, and DB86-CAH presented the typical semi-crystalline nature of cellulose hydrogel (Fig. 4b,c). Abroad peak observed with characteristic peaks at 2θ of 22°, 32.15°, 43.44°, and 45.88°, which show that the ionic liquid-induced destruction of cellulose's inter- and intramolecular hydrogen bonds caused the cellulose crystal shape to be disturbed.

#### SEM analysis

**Figure 5:**
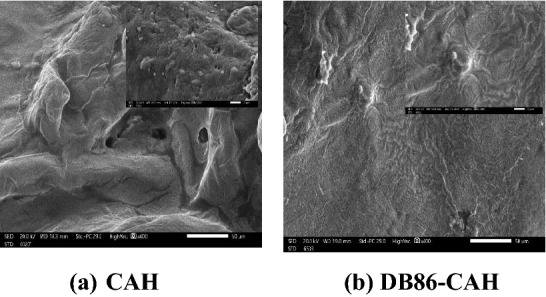
SEM images (**a**) CAH sample, (**b**) DB86-CAH sample.

The morphological features of CAH and DB86-CAH surfaces were examined by SEM study (Fig. 5). CAH demonstrated an apparent homogenous layer stacked and compact structure. This is in agreement with the reported literature^40,55^. After DB86 dye sorption, the surface morphology changes because the surface of CAH was filled with DB86 dye molecules.

Absorption of Direct Blue 86 dye on Cellulose Hydrogel.

#### Influence of pH

**Figure 6:**
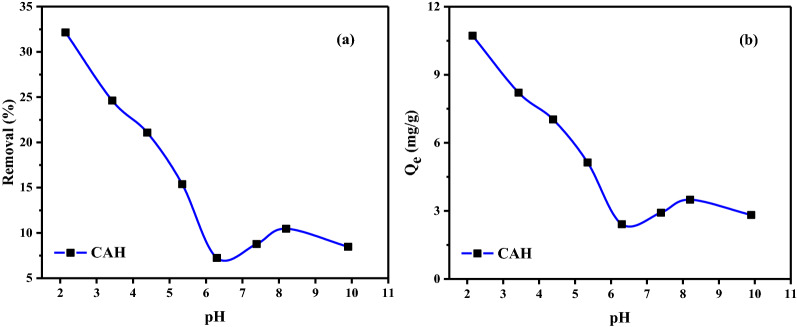
DB86 dye absorption on CAH as a pH impact (**a**) on the removal %; (**b**) on the absorption capacity [DB86 dye (50 ppm), adsorbent (1.5 g/L), time of contact (3 h), shacking (200 rpm), and temp. (27 ± 2 °C)].

Because of its impact on the CAH's surface characteristics and the ionization/dissociation of the adsorbate molecule, the solution pH likely has a serious impact on the adsorptive absorption of the molecules of absorbate^56^. At room temp. (25 ± 2 °C), with a beginning DB86 dye concentration of 50 mg/L and employing 1.5 g/L CAH as an absorbent, the quantity of Direct Blue 86 (DB86) dye absorbed at equilibrium (*q*_e_) and removal of this dye were determined. Figure 6 depicts the pH fluctuations over the three hours when the absorption of the DB86 dye was investigated at pH ranges of 2 to 10.

The maximum DB86 dye removal (32.15%) for the removal of DB86 dye using CAH occurred at pH 2, as can be observed in Fig. 6a. The absorption rate dropped from 32.15 to 7.23% in the investigation for the removal of the DB86 dye when the value of pH was steadily raised from 2 to 6. Then, there was a slight increase from pH 6 to 8, then a decrease towards pH 10, and reached around 8.46%. Figure 6b demonstrates that at pH 2, the maximum capacity is 10.72 mg/g, while at pH 6, the minimum absorption capacity is 2.41 mg/g.

This may be ascribed to the property of the produced CAH that caused it to attract hydrogen ions (H^+^) to the CAH surface when immersed in an acidic solution, making it positively charged and promoting the absorption of anionic samples of DB86 dye. Due to the CAH surface's positive charge at low pH levels, a very strong electrostatic attraction forms between the anionic dye molecule and the positively charged CAH surface, which promotes the most significant DB86 dye absorption. However, as the pH level (basic condition) rose, there were more negatively charged sites and fewer sites positively charged, resulting in a shift in the balance of charges. An anionic DB86 dye molecule cannot be attracted to a negatively charged CAH surface because of electrostatic attraction.

Similar effects of pH were reported for removing DB86 dye on AC produced from orange peels^56^, shrimp chitosan^57^, and prepared alginate encapsulated activated carbon^58^.

#### Time of contact influence

**Figure 7:**
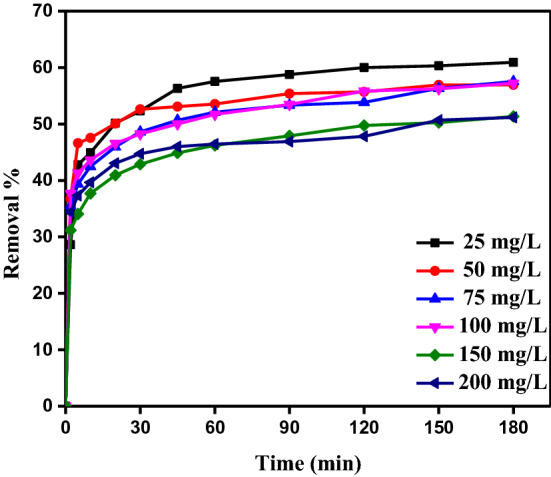
The absorption of DB86 dye for the 3 h by CAH (*C*_0_ of DB86 dye (25–200 mg/L), CAH dose (9.0 g/L), temp. (25 ± 2 °C).

At varying shaking times and concentrations of 25, 50, 75, 100, 150, and 200 mg/L, Fig. 7 illustrates the impact of contact time on the ability of CAH to bind the DB86 dye^5,33^. With an increase in absorption time and starting concentration, DB86 dye’s capacity for absorption also increases. Absorption capacity was found to rise during the first five minutes, after which it was discovered that the rate of absorption was slow. The reason for the rapid absorption during the starting contact period is the availability of the positively charged surface of the CAH for the absorption of DB86 dye at pH 2. The slower rate of DB86 dye absorption is likely caused by electrostatic resistance or repulsion between the absorbed negatively charged absorbate species on the surface of CAH and the available anionic absorbate species in solution as well as the slow pore diffusion of the solute ions into the bulk of the absorbent. The absorption equilibrium was basically achieved within 30 min for the initial DB86 dye concentration of 25–200 ppm.

#### Influence of beginning DB86 dye concentration

**Figure 8:**
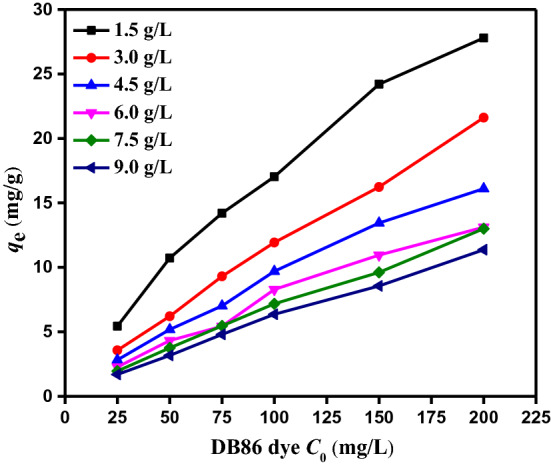
The impact of DB86 dye beginning concentration (25–200 mg/L) using CAH doses (1.5–9.0 g/L) on *q*_*e*_ (mg/g), temp. (25 ± 2 °C).

The capacity of absorption onto CAH was examined, and Fig. 8 illustrates the impact of the beginning DB86 dye concentration. At room temp. (25 ± 2 °C), the absorption studies were conducted at pH 2 and various starting DB86 dye concentrations (25, 50, 75, 100, 150, and 200 mg/L). The contact time was 180 min, and the CAH dose was 1.5–9.0 g/L in the test solution. The data showed that the CAH absorption efficiency percentage decreased as the starting concentration of DB86 dye in the solution increased, clearly demonstrating that absorption of DB86 dye from its aqueous solution was dependent on starting concentration and that an increase in DB86 dye concentration resulted in a reduction in the size of the active surface^56^. When the beginning concentration of DB86 dye increased from 25 to 200 mg/L, respectively, the elimination % of DB86 dye at doses 1.5 g/L and 9.0 g/L dropped from 32.62 to 20.85% and 60.92 to 51.15%. On the other hand, as the starting DB86 dye concentration rises, so does the absorption capacity. When the starting DB86 dye concentration is increased from 25 to 200 mg/L, respectively, the *Q*_m_ at doses of 1.5 g/L and 9.0 g/L rose from 5.44 to 27.79 mg/g and 1.69 to 11.37 mg/g.

#### Impact of CAH dosage on DB86 dye absorption

**Figure 9:**
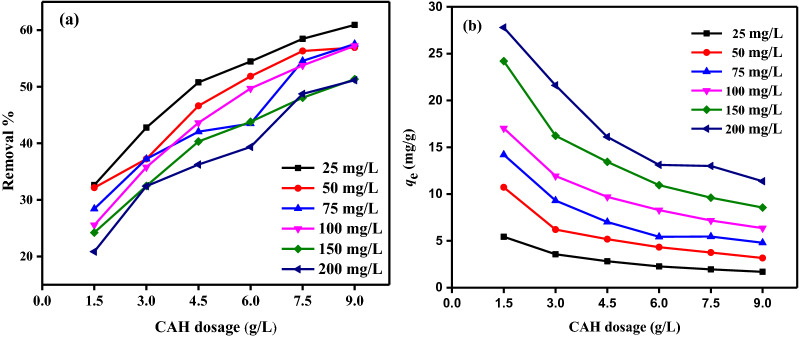
The influence of CAH doses (1.5–9.0 g/L) of various beginning DB86 dye concentrations (25–200 mg/L) on (**a**) % of removal; (**b**) *q*_e_ (mg/g), at temp. (25 ± 2 °C).

By adjusting the adsorbent CAH dose from 1.5 to 9.0 g/L and using various concentrations of the dye DB86 from 25 to 200 mg/L at pH 2, it was possible to determine the CAH dose impact on the absorption of the dye (Fig. 9). It has been noted that when CAH concentration increased, DB86 dye elimination also increased, reaching a high of 60.92% at 25 mg/L DB86 dye^44,56^. Thus, the equilibrium adsorption capacity (*q*_e_) of DB86 dye declines. This outcome can be explained by the fact that greater CAH doses offer more CAH functional groups, surface area, and pores volume that will be available for adsorbing DB86 dye on CAH surface, however, the amount of DB86 dye absorbed per unit of increased CAH dose decreased.

#### Temperature influence on the absorption of DB86 dye

**Figure 10:**
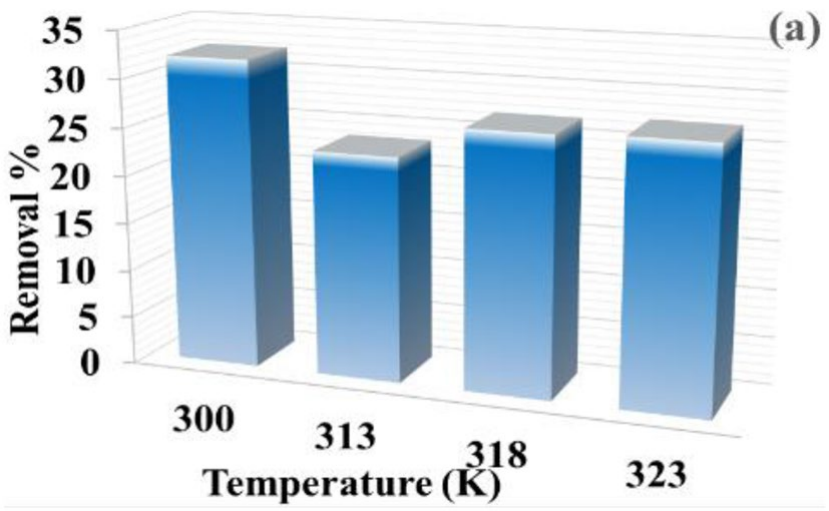
Temperature influence on the absorption capacity of CAH (1.5 g/L) at pH 2, DB86 dye (50 ppm) after 180 min.

Figure 10 depicts how temperature affected the absorption capacity in the 300–323 K temperature range. Increasing temperature led to a rapid decrease, then slightly increased the absorption capacity. By doing this, the rate of diffusion of the DB86 dye across the exterior boundary layer and within the cellulose hydrogel's interior pores is first decreased and subsequently increased^44,56^. For instance, with a starting concentration of the solution of 50 mg/L at around 323 K, pH 2, and 1.5 g/L CAH dosage, the highest equilibrium absorption capacity was 10.72 mg/g at temp. 300 K.

#### Absorption isotherms

Absorption isotherms models Langmuir (LIM), Freundlich (FIM), Temkin (TIM), and Dubinin–Radushkevich (DRIM) are used to describe the experimental result of DB86 on CAH. These isotherm models (IMs) are used to offer some information regarding the absorption mechanism, the surface characteristics, and the affinity of the absorbent^59^. The IMs were analyzed using error function analysis chi-square error (X^2^) to verify the validity of each linearized isotherm model and identify the best IM that represents the experimental results.

**Figure 11:**
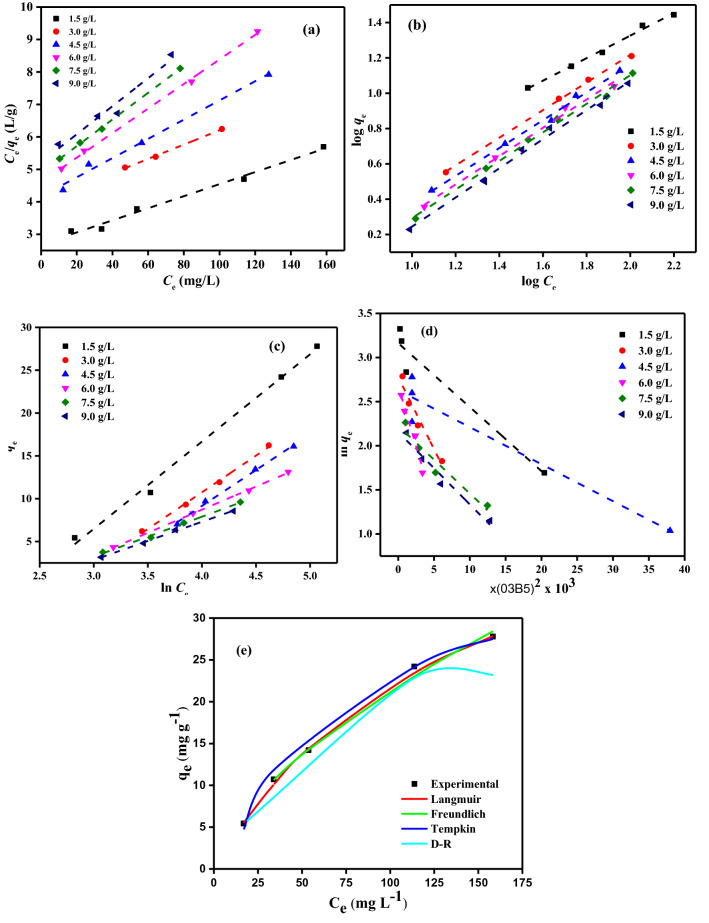
(**a**) LIM (**b**) FIM (**c**) TIM (**d**) DRIM profiles for DB86 dye of *C*_0_ (25–200 mg/L) on CAH (1.5–9.0 g/L) at (25 ± 2 °C) (**e**) Differentiation between the experimental results and estimated from IM for DB86 dye of *C*_0_ (25–200 mg/L) on CAH (1.5 g/L) at (25 ± 2 °C).

The LIM proposed that solute sorption from water solution occurs as monolayer absorption on specific homogenous sites with a finite number of identical sites. This LIM also assumes uniform absorption energies on the surface, no sorbate transmigration in the surface plane, and no interaction between adsorbed species^60–62^. Therefore, this data predicted the maximal absorption capacity of DB86, which corresponds to full monolayer coverage on the CAH sorbent surface. The linear mathematical expression of LIM is given by Eq. (3)^63^.

3 $$\frac{{C}_{e}}{{q}_{e}}=\frac{1}{{K}_{a}{Q}_{m}}+\frac{1}{{Q}_{m}}\times {C}_{e}$$

where *Q*_m_ (mg/g) is the maximum capacity, and *K*_*a*_ (L/mg) is the absorption equilibrium constant related to the apparent energy of sorption. The values of *Q*_m_ and *K*_a_ can be measured from the plot of *C*_e_ against *C*_e_/*Q*_e_ (Fig. 11a and Table 2). The *Q*_m_ was 53.76 mg/g at a CAH dose of 1.5 g/L.

**Table 2:** IMs investigation results of DB86 dye absorption by CAH [*C*_0_ (25–200 mg/L), CAH doses (1.5–9.0 g/L), and temp. (25 ± 2 °C)].

IMs	Parameters	CAH doses (g/L)					
		1.5	3.0	4.5	6.0	7.5	9.0
LIM	*Q*_m_ (mg/g)	53.76	45.45	33.78	26.46	24.33	23.15
	*K*_L_ × 10^3^	0.01	0.01	0.01	0.01	0.01	0.01
	*R*^2^	0.988	0.998	0.990	0.999	0.999	0.960
	X^2^	41.54					
FIM	*1/n*	0.64	0.78	0.79	0.80	0.81	0.83
	*Q*_m_ (mg/g)	33.00	79.97	29.93	18.46	22.19	20.84
	*K*_F_ (mg^1–1/n^ L^1/n^ g^–1^)	1.12	0.92	0.39	0.33	0.30	0.26
	*R*^2^	0.993	0.999	0.995	0.993	0.995	0.993
	X^2^	6.85					
TIM	*A*_*T*_	0.094	0.064	0.055	0.092	0.099	0.094
	*B*_*T*_	10.19	8.56	8.37	5.40	4.67	4.46
	*b*_*T*_ (J/mol)	244.87	291.39	298.16	461.96	534.24	558.77
	*R*^2^	0.995	0.997	0.997	0.999	0.996	0.998
	X^2^	0.00					
DRIM	*Q*_m_ (mol/kg)	23.64	16.08	13.88	14.57	9.27	8.63
	*K* × 10^3^ (mol/kJ)^2^	72.50	163.30	42.00	274.50	76.80	82.10
	*E* (kJ/mol)	2.63	1.75	3.45	1.35	2.55	2.47
	*R*^2^	0.941	0.946	0.928	0.966	0.929	0.962
	X^2^	0.03					

Meanwhile, FIM describes the absorption process based on heterogeneous surface sorption^64^. It is presumed that as the fraction of occupied sites increases, the enthalpy of sorption will decrease logarithmically. Equation (4) provides the linear mathematical formulas for FIM.

4 $$\mathrm{log}{q}_{e }={\mathrm{log}K}_{f }+\frac{1}{n}\mathrm{log}{C}_{e}$$

where *q*_e_ is the amount of DB86 dye adsorbed, ${K}_{f}$ (mg/g) is the FIM constant, and *n* is the heterogeneity factor that indicates the degree of non-linearity between solution concentration and absorption, which is determined from the slope of a plot between log *C*_e_ versus log *q*_e_^44^. The plot of the linear FIM for the absorption of the DB86 dye onto CAH is represented in Fig. 11b and Table 2. The value of *n* was more than 1, demonstrating that the absorption process is advantageous, with higher values of *n* demonstrating a more vital interaction between the adsorbate-adsorbent. Absorption is a beneficial physical process where the value of *n* is greater than unity^65^.

TIM is used to evaluate the absorption potentials^66^. A homogeneous allocation of binding energies, up to a maximum binding energy, is thought to describe absorption, and it is hypothesized that the heat of absorption of all the molecules in the layer reduces linearly with coverage due to the interactions between the adsorbate–adsorbate. The TIM linear version has been used as in Eq. (5).

5 $${q}_{e}=B \mathrm{ln} A+B \mathrm{ln}{C}_{e}$$

where* B* (*RT*/*b*) is TIM constant and related to the heat of sorption since *T* (kelvin) is the absolute temp. and *R* (8.314 J/mol.K) is the universal gas constant. *A* (L/g) is the equilibrium binding constant related to the maximum binding energy^44^. Plotting ${q}_{e}$ versus ln ${C}_{e}$ according to Eq. (5) enables us to determine the TIM constants *A* and *b,* (Fig. 11c). The IM results calculated from TIM are presented in Table 2. The heat of DB86 dye removal by CAH was found to increase from 244.87 to 558.77 J/mol with the increase of CAH dose from 1.5 to 9.0 g/L.

Dubinin–Radushkevich isotherm model (DRIM) was also used to predict the porosity apparent free energy and the characteristics of the removal process^67–69^. It may be applied to explain absorption on both heterogeneous and homogenous surfaces, and it has been applied in the following Eq. (6)^70^:

6 $$\mathrm{ln}{q}_{e}=\mathrm{ln}{Q}_{m}-K{\varepsilon }^{2}$$

where *Q*_m_ is the theoretical saturation capacity, *K* is an absorption energy-related constant, and is the Polanyi potential that may derive from Eq. (7):

7 $$\varepsilon =RT\mathrm{ln}\left(1+\frac{1}{{C}_{e}}\right)$$

Plotting *ε*^2^ versus ln *q*_e_ (Fig. 11d), enable to determination of *K* (mol^2^/kJ^2^) and the absorption capacity* Q*_m_ (mg/g) can be measured from the intercept value. The mean free energy of absorption is the change in free energy that happens when one mole of ions is transported from infinity (*E*) in solution to the surface of the sorbent. Using the following relation in Eq. (8), *E* was computed from the *K* value.

8 $$E=\frac{1}{\sqrt{2K}}$$

DRIM constants and *R*^2^ measured are shown in Table 2. The *Q*_m_ estimated for absorption of DB86 dye is 23.64 mg/g, which is lower than *Q*_m_ calculated from the LIM. The calculated *E* values are 1.35–3.45 kJ/mol, which is below 8 kJ/mol proposing that the physico-sorption process plays an important role in the absorption of DB86 dye by CAH.

On the basis of the error analysis function (chi-square error, X^2^) to measure the goodness-of-fits IM, it can assume that the TIM is the best IM for the adsorptive removal of DB86 dye by CAH (Fig. 11e).

#### Absorption kinetic studies

The kinetic models (KMs) results of DB86 dye absorption by CAH were studied to realize the mechanism of absorption. Five KMs were employed including, Elovich (EM), pseudo-first-order (PFOM), pseudo-second-order (PSOM), liquid film diffusion (LFD), and intraparticle diffusion (IPDM) models. The identified parameters for the DB86 dye adsorptive removal by CAH were shown in Tables 3 and 4. The *R*^2^ (values close or equal to 1) represented the degree of agreement between experimental data and model-predicted values.

**Table 3:** PFOM and PSOM results of AB86 dye absorption [*C*_0_ (25–200 mg/L) by CAH doses (1.5–9.0 g/L), and Temp. (25 ± 2 °C)].

Parameter		PFOM				PSOM			
CAH (g/L)	DB86 dye (mg/L)	*q*_*e*_ (exp.)	*q*_*e*_ (calc.)	*k*_*1*_ × 10^3^	*R*^2^	*q*_*e*_ (calc.)	*k*_*2*_ × 10^3^	*h*	*R*^2^
1.5	25	5.44	1.96	15.20	0.916	5.52	28.64	0.87	0.996
	50	3.56	1.89	16.35	0.855	10.71	41.52	4.76	1.000
	75	2.82	4.59	21.65	0.953	14.37	15.56	3.21	0.999
	100	2.27	5.34	21.65	0.988	17.24	13.09	3.89	0.999
	150	1.95	8.63	28.33	0.744	24.15	12.60	7.35	0.999
	200	1.69	10.38	14.28	0.841	27.55	5.79	4.39	0.994
3.0	25	10.72	1.36	19.81	0.934	3.60	48.81	0.63	0.997
	50	6.21	2.95	26.95	0.858	6.26	30.68	1.20	0.998
	75	5.18	4.51	30.63	0.824	9.42	22.40	1.99	0.999
	100	4.32	4.67	18.42	0.883	11.99	13.59	1.95	0.996
	150	3.75	5.62	21.42	0.920	16.50	10.78	2.94	0.996
	200	3.16	6.62	31.32	0.927	21.88	15.76	7.55	1.000
4.5	25	14.21	1.23	24.87	0.974	2.88	58.60	0.49	0.998
	50	9.31	2.60	32.93	0.776	5.23	44.02	1.20	0.999
	75	7.01	2.31	21.19	0.960	7.10	30.90	1.56	0.999
	100	5.44	3.60	23.26	0.895	9.79	21.03	2.02	0.998
	150	5.46	5.95	30.63	0.866	13.61	16.79	3.11	0.999
	200	4.79	4.76	16.12	0.972	16.16	13.83	3.61	0.999
6.0	25	17.03	1.22	30.86	0.838	2.33	67.30	0.36	0.999
	50	11.92	1.34	24.18	0.990	4.38	61.37	1.18	1.000
	75	9.69	1.77	28.79	0.930	5.49	58.29	1.76	1.000
	100	8.28	2.80	17.27	0.984	8.34	22.93	1.59	0.998
	150	7.17	3.60	19.58	0.993	11.07	19.05	2.34	0.999
	200	6.36	6.25	28.56	0.802	13.19	15.26	2.66	0.998
7.5	25	24.21	1.04	29.94	0.906	1.99	87.56	0.35	0.999
	50	16.23	1.06	22.57	0.973	3.81	72.44	1.05	0.999
	75	13.44	1.72	18.19	0.973	5.50	40.04	1.21	0.999
	100	10.95	3.65	32.24	0.760	7.22	29.89	1.56	0.999
	150	9.61	3.04	18.19	0.983	9.69	22.42	2.10	0.999
	200	8.56	4.05	17.73	0.960	13.05	16.66	2.84	0.998
9.0	25	27.79	0.56	26.02	0.968	1.72	149.22	0.44	1.000
	50	21.64	0.96	21.42	0.909	3.19	124.11	1.26	1.000
	75	16.10	1.47	16.81	0.941	4.80	45.92	1.06	0.998
	100	13.12	2.95	3.16	0.803	6.42	35.49	1.46	0.999
	150	12.99	3.89	28.10	0.849	8.64	24.21	1.81	0.999
	200	11.37	8.61	34.78	0.742	11.36	22.68	2.93	0.998

**Table 4:** EM, IPDM and FDM results of DB86 dye absorption [*C*_0_ (25–200 mg/L) by CAH doses (1.5–9.0 g/L), and Temp. (25 ± 2 °C)].

Parameter		EM			IPDM			FDM		
CAH (g/L)	DB86 dye (mg/L)	*Β*	*Α*	*R*^2^	*К*_*dif*_	*C*	R^2^	*К*_*FD*_	*C*	*R*^2^
1.5	25	1.82	5.51E + 01	0.973	0.19	2.91	0.920	3.41E−02	0.462	0.840
	50	1.57	7.62E + 04	0.950	0.21	7.96	0.845	2.80E−02	1.331	0.760
	75	0.97	4.89E + 03	0.971	0.36	9.31	0.977	3.05E−02	0.825	0.868
	100	0.84	8.50E + 03	0.929	0.43	11.25	0.967	3.14E−02	0.828	0.865
	150	0.64	4.23E + 04	0.989	0.53	16.96	0.936	2.83E−02	1.032	0.744
	200	0.36	2.76E + 02	0.956	0.94	14.53	0.904	1.42E−02	0.985	0.841
3.0	25	2.89	5.19E + 01	0.986	0.12	1.97	0.928	1.97E−02	0.959	0.934
	50	1.67	1.04E + 02	0.997	0.20	3.50	0.931	2.70E−02	0.746	0.858
	75	1.22	3.73E + 02	0.994	0.28	5.57	0.953	3.06E−02	0.726	0.824
	100	0.93	2.85E + 02	0.980	0.38	6.69	0.965	1.84E−02	0.938	0.883
	150	0.81	2.62E + 03	0.880	0.44	10.15	0.913	2.14E−02	1.060	0.920
	200	0.66	1.54E + 04	0.991	0.51	15.17	0.907	3.13E−02	1.184	0.927
4.5	25	3.52	3.22E + 01	0.980	0.01	1.52	0.962	2.49E−02	0.831	0.974
	50	2.19	2.23E + 02	0.990	0.16	3.14	0.932	1.92E−02	1.166	0.974
	75	1.64	3.36E + 02	0.978	0.21	4.25	0.943	2.12E−02	1.109	0.960
	100	1.22	5.84E + 02	0.996	0.28	5.93	0.948	2.32E−02	0.991	0.895
	150	0.93	1.59E + 03	0.988	0.37	8.51	0.966	2.13E−02	1.139	0.994
	200	0.76	1.39E + 03	0.992	0.45	10.01	0.945	1.62E−02	1.218	0.972
6.0	25	2.98	2.36E + 00	0.833	0.11	0.97	0.658	3.08E−02	0.624	0.838
	50	2.64	2.35E + 02	0.993	0.13	2.72	0.896	2.41E−02	1.174	0.990
	75	2.23	5.94E + 02	0.972	0.15	3.58	0.851	2.88E−02	1.125	0.930
	100	1.34	2.35E + 02	0.994	0.26	4.81	0.945	1.72E−02	1.083	0.984
	150	1.07	5.92E + 02	0.993	0.32	6.65	0.958	3.29E−02	0.654	0.790
	200	0.92	9.16E + 02	0.989	0.37	8.02	0.950	2.86E−02	0.742	0.802
7.5	25	4.13	5.21E + 00	0.962	0.08	0.93	0.848	2.31E−02	0.866	0.955
	50	3.29	4.59E + 02	0.995	0.10	2.45	0.916	2.25E−02	1.260	0.973
	75	2.06	2.02E + 02	0.990	0.17	3.27	0.934	1.82E−02	1.153	0.973
	100	1.77	8.54E + 02	0.992	0.20	4.51	0.968	1.81E−02	1.165	0.988
	150	1.21	4.79E + 02	0.972	0.29	5.81	0.936	1.83E−02	1.152	0.983
	200	0.92	8.57E + 02	0.994	0.37	8.01	0.929	1.78E−02	1.165	0.960
9.0	25	5.43	1.45E + 01	0.946	0.06	0.96	0.796	2.59E−02	1.102	0.968
	50	4.57	3.10E + 03	0.927	0.07	2.28	0.779	2.14E−02	1.573	0.909
	75	2.47	2.80E + 02	0.994	0.14	2.93	0.933	1.67E−02	1.183	0.941
	100	2.05	1.09E + 03	0.993	0.17	4.08	0.966	1.94E−02	1.187	0.985
	150	1.30	2.86E + 02	0.997	0.26	5.07	0.944	1.89E−02	1.106	0.985
	200	1.24	5.22E + 03	0.982	0.27	7.63	0.912	1.81E−02	1.275	0.850

**Figure 12:**
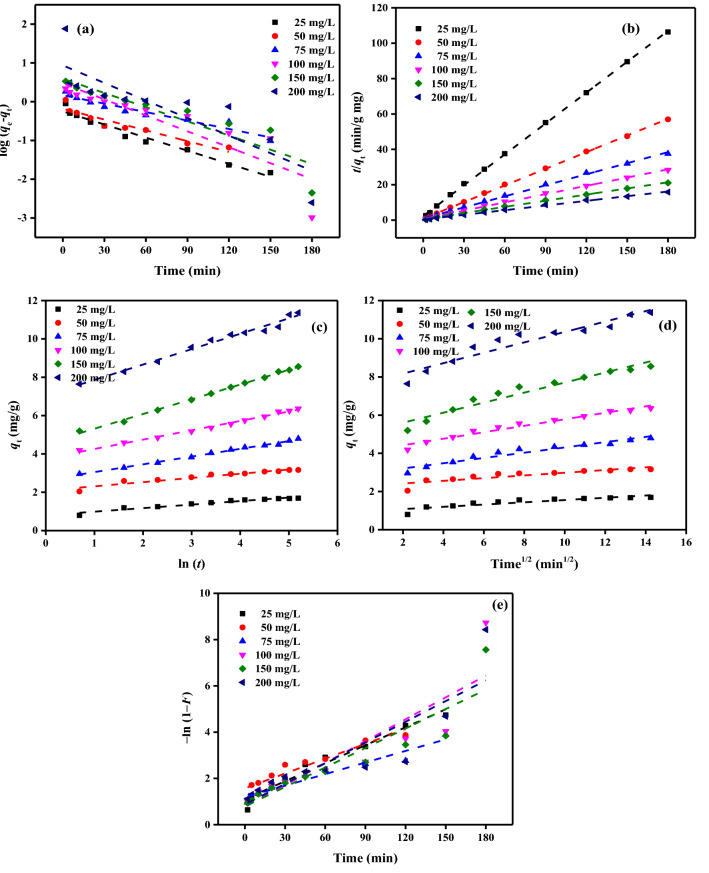
(**a**) PFOM, (**b**) PSOM, (**c**) EM, (**d**) IPDM, (**e**) FDM of absorption of DB86 dye [*C*_0_ (25–200 mg/L) by CAH dose (9.0 g L^−1^), temp. (25 ± 2 °C)].

The PFOM^71^, the first reported model, studied the sorption rate based on the capacity of sorption. The linear PFOM is expressed by Eq. (9):

9 $$\mathrm{log}({q}_{e}-{q}_{t})=\mathrm{log} ({q}_{e})-\frac{{k}_{1}}{2.303}t$$

where *q*_t_ and *q*_e_ (mg/g) are the absorption capacity at time *t*, and at equilibrium, respectively, *k*_1_ (L/min) is the rate constant of PFOM (Fig. 12a).

From Table 3, the experimental *q*_e_ does not agree with the predicted *q*_e_ values, and the correlation coefficient *R*^2^ is relatively low, so it has not followed PFOM.

PSOM was investigated to study the nature of binding between DB86 dye and CAH either chemical or physical absorption. The linear PSOM is shown by Eq. (10)^72^.

10 $$\left(\frac{t}{{q}_{t}}\right)=\frac{1}{{k}_{2}{q}_{e}^{2}}+\frac{1}{{q}_{e}}\left(t\right)$$

The beginning sorption rate, *h* (mg/g.min) is calculated using Eq. (11):

11 $$h={k}_{2}{q}_{e}^{2}$$

where *k*_2_ (g/mg. min) is the pseudo-second-order constant (Fig. 12b).

From Table 3, the experimental *q*_e_ agrees with the predicted *q*_e_ values, and the correlation coefficients *R*^2^ are greater than 0.990, so it followed PSOM.

The EM is a rate equation based on the absorption capacity. The linear form of EM is generally expressed by Eq. (12)^73–75^.

12 $${q}_{t}=\frac{1}{\upbeta }\mathrm{ln}(\mathrm{\alpha \beta } )+ \frac{1}{\upbeta }\mathrm{ln}\left(t\right)$$

where *α* (mg/g.min) is the starting absorption rate, and *β* (g/ mg) is the desorption constant (Fig. 12c).

From Table 4, the correlation coefficients measured by EM ranged from (0.833–0.997), the *α*, has a wavy and unidentified role with DB86 dye concentration and CAH dose. The *β* decreases with increasing the beginning DB86 dye concentration while increasing with an increased CAH dose.

The linear form of IPDM was generally expressed by Eq. (13)^76,77^. The solute molecules are transferred from the water medium to the surface of the adsorbent as the first step in the mechanism of the intraparticle diffusion model. Next, the adsorbate molecules are diffused into the pores of the CAH (slow process).

13 $${q}_{t}= {K}_{dif}{t}^{1/2}+C$$

where *C* (mg/g) is the intercept and *K*_dif_ (mg/g.min^1/2^) is the IPDM rate constant, which is related to the boundary layer thickness (Fig. 12d).

From Table 4, the *R*^2^ calculated for the IPDM ranged from 0.658 to 0.977. Although it was a part of the process, the IPDM plots show that IPD was not the only phase controlling the rate. Directly proportional to DB86 dye concentration and inversely proportional to CAH dosages are the values of *K*_dif_ and C.

The values of *C* were found to be inversely proportional to CAH doses, reflecting a decrease in boundary layer thickness and an increase in the chance of external mass transfer. The values of *C* were also found to be directly proportional to DB86 dye concentration, indicating an increase in boundary layer thickness and a decrease in the opportunity for external mass transfer, and an increase in the chance of internal mass transfer. The *K*_dif_ values were in the range of 0.0595–0.939 mg/g min^1/2^ and were found to increase with an increase in DB86 dye concentration and decrease with increased CAH doses.

Finally, the kinetics of DB86 dye absorption by CAH were studied using FDM^78^. When solute molecules are transported from the liquid phase to the solid phase, boundaries play a crucial role in absorption. This FDM was given by Eq. (14):

14 $$\mathrm{ln}\left(1-F\right)=-{K}_{FD}\left(t\right)$$

where *F* (*F* = *q*_t_/*q*_e_) and *K*_FD_ are the fractional attainments of equilibrium and the FD rate constant, respectively (Fig. 12e).

From Table 4, the *R*^2^ obtained by the liquid film diffusion model ranged from 0.744—0.994. The linearity of the FDM plots, which do not pass via the origin, demonstrates how the absorption rate is impacted by the FD mechanism. The relationship between the values of *K*_FD_ and *C* and the doses of CAH and DB86 dye is wavy and ambiguous.

#### Absorption thermodynamics

Results from experiments of DB86 dye absorption by CAH at various temperatures were used to estimate the values of thermodynamic constants such as enthalpy changes (Δ*H*°), Gibbs energy changes (Δ*G*°), and entropy changes (Δ*S*°). They were measured using the following Eqs. (15, 16, 17):

15 $${\Delta G}^{o}=-{RT lnK}_{c}$$

16 $${InK}_{c}=\frac{{\Delta S}^{o}}{R}-\frac{{\Delta H}^{o}}{RT}$$

17 $$\Delta G=\Delta H-T\Delta S$$

where *K*_c_ (*K*_c_ = *q*_e_/*C*_e_, L/g) is the distribution coefficient, *R* (8.314 J/mol K) is the gas constant, and *T* (kelvin) is the absolute temperature. The calculated values for the changes of Δ*G*°, Δ*H*°, and Δ*S*° are shown in Table 5. All *∆G°* values were positive and increased with increasing temperature, indicating that the DB86 dye process of absorption on CAH was nonspontaneous. The negative *∆H°* value (9.36 kJ/mol) of DB86 dye absorption on CAH confirmed the involvement of an exothermic process. The entropy (*∆S°*) value was also negative (–40.84 J/K mol), which explains less disorder at the solid/solution interface during the DB86 dye absorption by CAH.

**Table 5:** Thermodynamic constants for the DB86 dye absorption at various temperatures on CAH.

Temp. (K)	*ΔG°* (kJ/mol)	*ΔH°* (kJ/mol)	*ΔS°* (J/K mol)
300	2.89	−9.36	−40.84
313	3.42		
318	3.63		
323	3.83		

#### Regeneration of CAH

**Figure 13:**
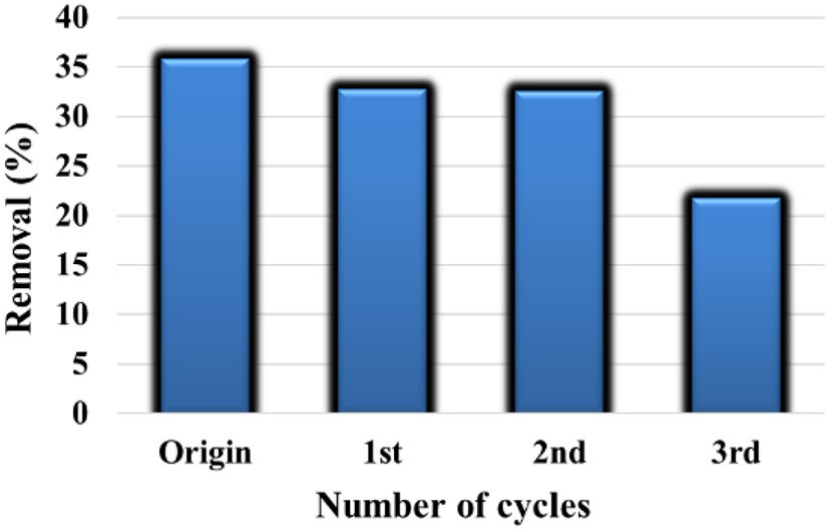
Removal % of DB86 dye by regenerated CAH.

To create sorption media for DB86 elimination that is cost-effective. For more cycles, CAH should be recycled^72^. Three successive cycles of the DB86-CAH regeneration process were carried out. Every cycle involved shaking the sorbent (10 g of CAH) with 100 mL of DB86 dye solution (100 mg/L) for three hours. Following effective sorption, the DB86 dye was eluted with a NaOH solution (0.1 mol/L, 100 mL) for 2.0 h before being cleaned with DW till the neutralization for reuse. Prior to and following the recovery procedure, the absorption of the DB86 dye by absorption on the adsorbent CAH was studied under similar conditions: *C*_0_ of 100 mg/L at about 25 °C, pH 2, and CAH dose of 3.0 g/L adsorbent. The maximum removal % of DB86 dye onto CAH before the recovering process was 35.77%, while after recovering process was 32.77%. Figure 13 illustrates the regeneration efficiency of DB86-CAH for the adsorptive of DB86 dye from water. It was observed that there is a slight decrease in the absorption capacity for DB86 dye after two cycles. These results confirm the possibility of the reuse of the sorbent.

#### Absorption mechanism

**Figure 14:**
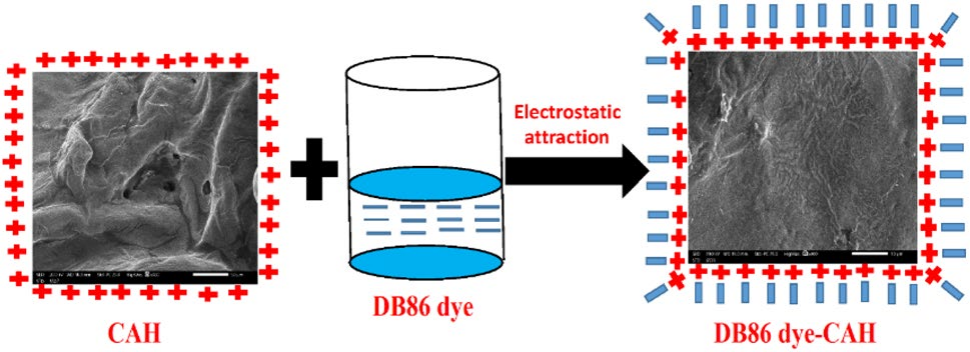
DB86 dye absorption mechanism on CAH.

The possible interactions between DB86 dye and cellulose hydrogel are shown in Fig. 14. The new hydrogel CAH is positively charged by nature at acidic pH values, which allows it to attract hydrogen ions (H^+^) onto its surface and favors the absorption of anionic samples of DB86 dye. The anionic dye molecule and positively charged surface provide a potent electrostatic attraction, which promotes the greatest amount of DB86 dye absorption. However, a basic pH value causes the surface of CAH to become negatively charged as data of the absorption of hydronium ions (OH^–^) to its surface, which makes anionic DB86 dye molecules less likely to adhere due to electrostatic repulsion^79,80^.

#### Comparison of findings with those documented in the literature

The CAH absorption capacity for DB86 dye was compared with different adsorbents (Table 6). The *Q*_m_ (53.76 mg/g) predicted for CAH was more than or comparable to that reported in the literature using various materials as adsorbents for the treatment of DB86 dye.

**Table 6:** Results of the *Q*_m_ and removal % of DB86 dye of various adsorbents reported in the literature.

Adsorbent name	Removal %	*Q*_m_ (mg/g)	IMs	KMs	Enthalpy	Ref erences
Aspergillus flavus A5p1	100	–	–	–	–	^81^
Shrimp chitosan continuous	100	–	–	–	–	^57^
Manioc husk activated carbon Commercial brand activated carbon	– –	6.1 3.7	–	PSOM	–	^82^
PnsAC-alginate	98.4	21.6	LIM	PSOM with IPDM and FDM	Endothermic	^58^
UVA/ZnO UVA/TiO2	69 95.5	– –	– –	– –	– –	^83^
Orange peel activated carbon	92	33.78	LIM	PSOM	–	^56^
Raw carbon nanotube modified carbon nanotube	–	69.93 149.25	LIM	PSOM	–	^84^
CrossPANI/P-AC(1:0.2)	–	163.93	LIM	PSOM	Endothermic	^85^
Cellulose hyrogel	60	53.76	TIM	PSOM	Endothermic	This study

## Conclusion

LiCl/Dimethylacetamide was used to generate cellulose hydrogel (CAH), which was then examined utilizing FT-IR, SEM, XRD, and TGA studies. In order to absorb the DB86 dye from an aqueous solution, batch equilibrium was used to test CAH's performance. Temkin's model was the best fit for the data after it was assessed using the LIM, FIM, TIM, and DRIM models. PFOM, PSOM, EM, IPDM, and FDM models were applied to analyze the kinetic adsorption results, and the PSO rate model was used to control the absorption rate. According to the thermodynamic characteristics, the DB86 dye absorption by CAH was exothermic and nonspontaneous. The regeneration results demonstrate the CAH's potential for reuse and its promise for removing the DB86 dye from water.

## Data Availability

The datasets used in this investigation are accessible for review upon request from the corresponding author of the paper.
